# The Goal Scale: A New Instrument to Measure the Perceived Exertion in Soccer (Indoor, Field, and Beach) Players

**DOI:** 10.3389/fpsyg.2020.623480

**Published:** 2021-01-07

**Authors:** Luis Felipe Tubagi Polito, Marcelo Luis Marquezi, Douglas Popp Marin, Marcelo Villas Boas Junior, Maria Regina Ferreira Brandão

**Affiliations:** ^1^Post-graduate Program in Physical Education, São Judas Tadeu University, São Paulo, Brazil; ^2^Physical Education Research Laboratory, Universidade Cidade de São Paulo, São Paulo, Brazil; ^3^Physical Education School, Methodist University of São Paulo, São Bernardo do Campo, Brazil

**Keywords:** psychophysiology, athletic performance, effort, fatigue, differentiated RPE

## Abstract

The rating of perceived exertion (RPE) can be used to monitor the exercise intensity during laboratory and specific tests, training sessions, and to estimate the internal training load of the athletes. The aim of the present study was to develop and validate a specific pictorial perceived exertion scale for soccer players (indoor, field, and beach soccer) called GOAL Scale. The pictorial GOAL Scale (six drawings; 1 “low exertion” to 6 “exhaustion”) was validated for twenty under-17 soccer players (16.4 ± 0.68 years; 175.4 ± 9 cm; 66.4 ± 7.7 kg; % fat mass 12.4 ± 3.3). In the validation phase, the athletes were evaluated in a progressive protocol involving stimuluses of 3 min with 1 min for the rest into the stages until the voluntary exhaustion in Maximal Cardiopulmonary Effort Test (MCET), and in the Yo Yo Intermittent Recovery Test – Level 1 (Yo-Yo). The RPE identified by the GOL Scale, by the Borg Scale 6 – 20 and by the Cavasini Scale, as well as the heart rate (HR), perceptual of the heart rate (%HR_max_) and the blood lactate concentration ([La]) were immediately evaluated after each stage of both tests. Spearman’s correlation coefficient (*p* < 0.05) was used. Construct scale validity was examined by regressing GOAL Scale against Borg Scale 6 – 20 and Cavasini Scale and concurrent scale validity was investigated by regressing GOAL Scale against HR, beats/min and blood lactate concentration (mmol/L) during two progressive tests. There was a significant correlation values of the GOAL Scale with Borg Scale (*r* = 0.93; *r* = 0.88), Cavasini Scale (*r* = 0.91; *r* = 0.90), %HR_max_ (*r* = 0.91; *r* = 0,86), HR (*r* = 0.87; *r* = 0.83) and lactate (*r* = 0.68; *r* = 0.83) during tests (Maximal Incremental Cardiopulmonary Test and Yo-Yo test, respectively). The results evidenced concurrent and construct validity of the GOAL Scale across a wide range of exercise intensity. The absence of verbal anchors makes the use of this instrument to soccer, futsal and beach soccer athletes of different languages and different literacy levels possible.

## Introduction

In general, the sports performance has been studied for researchers of different areas, once the systematic planning of athlete training programs is supposed to integrate physiological, biomechanical, nutritional, psychological and social dimensions (Mujika et al., 2018).

From the physical perspective, the increase of athlete’s performance of different sports, including futsal, beach soccer, and field soccer athletes shows a straight relationship with the proper control of the training units, including volume, intensity, and density of training (relation between total volume and session time) (Veugelers et al., 2016; Ribeiro et al., 2020). It is possible to affirm that this control is a great challenge for the physical trainers, once the low stimulus is not enough to generate positive adaptations of performance, while the excessive and rapid increases in training loads can contribute to the increase of the impairments (Gabbett, 2016).

Thus, the measure of the parameters which indicate the intensity of effort in which the athletes are submitted during the routine of training is necessary. These parameters can be analyzed by the physiological variables such as the oxygen consumption (VO_2_), the heart rate (HR), the ventilator anaerobic thresholds, blood lactate concentration (Bellotti et al., 2013), and by psychophysiological variables such as the ratings of perceived exertion (RPE) (Borg, 1962), the total quality recovery (McGuigan, 2017), Hooper index (Hooper and Mackinnon, 1995), and the questionnaire of training load evaluation in soccer (Rebelo et al., 2012).

According to Borg (1962); Noble and Robertson (1996), and Borg and Kaijser (2006), the RPE is a variable frequently used in physical exercise ambit and, more specifically, in sports because it has a great correlation with other exertion and objective parameters, such as heart rate and blood lactate. According to Impellizzeri et al. (2005) the RPE is a variable frequently used in the soccer as well, as it makes it possible for coaches and physical trainers to evaluate the intensity of the exercises exerted in a training session.

Recently, Thorpe et al. (2016) highlighted the importance to the use of psychometric instruments in training routine (scales and questionnaires), and they were able to show that the recovery was better identified by the Total Quality Recovery (TQR) scale rather than by the heart rate variability, a gold standard parameter used to evaluate recovery in sports. The former is a subjective method while the latter is an objective one but it requires more complexity as more costly instruments are involved, limiting its application in clubs from different economic backgrounds.

Based on this reality, the literature reveals that both the Borg RPE scale Borg (1962, 1998) and the Borg CR10 Scale adapted by Foster et al. (2001) are the most frequently used instruments for the effort measurement and training load in soccer and futsal athletes (Moreira et al., 2013; Polito et al., 2017). It is important to highlight here that the Foster’s Scale was not validated using the psychophysical principles, which can be observed by the use of the “Maximal” anchor at number 10, which introduces a ceiling effect that roots out the scale properties.

However, it is important to highlight that the RPE instruments cited were created and validated to be used for all kind of sports and even several kinds of perceptions, and symptoms. So, these instruments do not take in account the specificity of the athletes, including soccer players. Besides that, a very common problem is that the translations of the original instrument have been done without proper cross-cultural adaptation processes, which contributes with the same instrument using different verbal anchors for the same level of the scale. Other limitation of the verbal scales is that not all individuals are able to interpret verbal anchorages adequately, which may be related to the level of literacy of the evaluated subjects.

Following this train of thought, Bar-Or and Ward (1989), Robertson et al. (2000); Kang et al. (2003), Utter et al. (2006), and Chen et al. (2017), argued that scales consisting solely of pictorial images are easier to understand to apply in different groups, which may minimize the limitations in the interpretation of psychophysiological perception and its evaluation. In addition to this, the ecological validity is increased when instruments and assessments comprehend biocultural manifestations of the specific context of the sport (Tenenbaum and Bar-Eli, 1995; Yelling et al., 2002; McGuigan, 2017).

The initial hypothesis was which the GOAL scale has high correlation levels with the physiological and psychophysical indicators, being then an alternative to identify the perceived exertion in soccer athletes without the risk of semantic problems found in the verbal scales. Therefore, the objectives of the present research were: (1) to elaborate a specific pictorial scale of the perceived exertion for soccer athletes called GOAL Scale; (2) to explore the association between the GOAL Scale and physiological indicators (heart rate – HR, percentage of maximal heart rate – % HRmax, oxygen consumption – VO_2_, percentage of maximum oxygen consumption – VO_2max_, blood lactate concentration); (3) to explore the association between the GOAL Scale and the other constructs already validated for the RPE measurement (Borg RPE 6–20 and Cavasini Scale). Cavasini Scale was validated for Brazilian people, consisting of 11 numeric anchorages and just 2 verbal anchorages (Cavasini and Matsudo, 1993).

## Materials and Methods

### General Design

**FIGURE 1:**
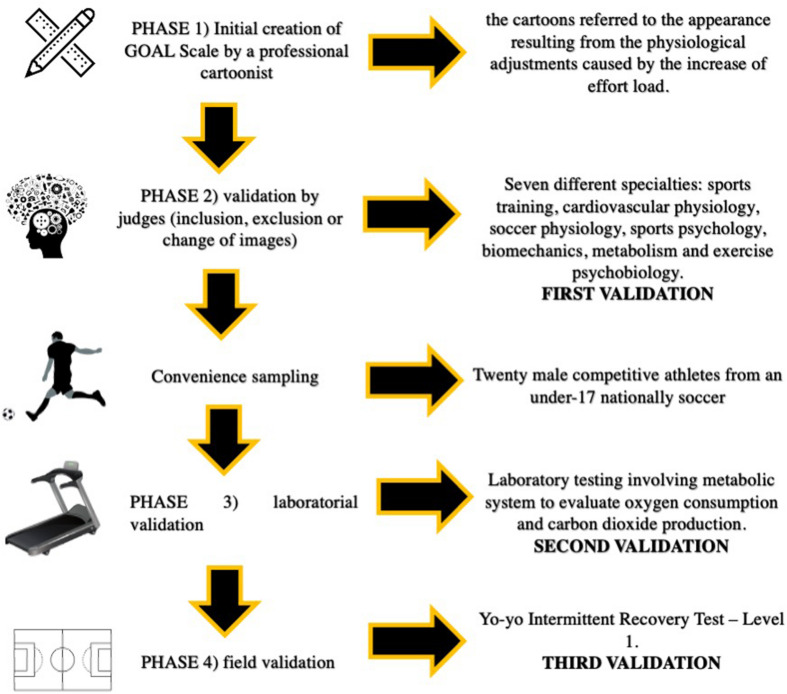
Schematic figure of the experimental design.

The present experimental research occurred in four steps as follow: (1) initial creation of GOAL Scale by a professional cartoonist; (2) validation by judges; (3) laboratorial validation; (4) field test validation (Figure 1).

The GOAL Scale was originally developed by a professional cartoonist. Initially, it was constituted of six images and their respective numbers (1 to 6), without any verbal anchorage. The initial requirements for the drawing of the cartoons referred to the appearance resulting from the physiological adjustments caused by the increase of effort load during the practice of the activity, including changes in the rate of sweating, facial flushing, and hyperventilation. The initial criteria established by the authors considered two images for each exercise phase, according to the multiple threshold model proposed by Skinner and McLellan (1980). In the second phase, the instrument was sent to nine Ph.D. professors, from seven different specialties for evaluation and positioning: sports training, cardiovascular physiology, soccer physiology, sports psychology, biomechanics, metabolism, and exercise psychobiology. After receiving the initial instrument, each judge had a period of 3 months to suggest the inclusion’ of new images, the exclusion of one or more of the six initially elaborated or the change of any details in any existing image of the instrument.

Thereafter, the GOAL Scale was tested in two different cardiorespiratory maximal tests: laboratory testing involving metabolic system to evaluate oxygen consumption and carbon dioxide production, and a field test which was validates to be applied in soccer players, the Yo-yo Intermittent Recovery Test – Level 1 (Castagna et al., 2006). The interval between the two tests was 1 week.

In order to have a better control of the test, the following measures were adopted: (1) familiarization of researchers and athletes in both tests, in which all instruments were introduced. The familiarization was done twice, 1 week before the first real test; (2) assessment in a 3-h postprandial state and (3) athletes were instructed not to use diuretics during 7 days before the test and not to ingest alcoholic drinks, coffee, energy drinks, and tea on the previous day of the test; (4) it was suggested that the subjects should urinate 30 min before the test. The laboratory and field tests were performed with a minimum interval of 48 h.

### Ethical Approval

The risks and benefits of the research were put on view, and each subject signed the Free and Informed Consent Term (FICT) and, in the case of children under the age of 18, the Free and Informed Assent Term (FIAT). The experimental model was approved by the Ethics Committee of São Judas University – protocol number 37500414.4.0000.0089.

### Participants

Twenty male competitive athletes from an under-17 national soccer team were analyzed during the competitive period. The evaluated subjects had a minimum experience of 3 years in the modality and the average daily training time of 2 h. The average age was 16.40 ± 0.68 years; stature: 175.45 ± 9.0 cm; total body mass: 66.4 ± 7.75 kg; percentage of body fat: 12.4 ± 3.31%; fat free mass: 58.2 ± 7.30 kg; maximum oxygen consumption of 60.8 ± 4.36 ml/kg/min.

The inclusion criteria were: (1) to practice competitive soccer, with a minimum of 1 h 30 m training per day, 5 days a week; (2) to have a 3-year minimum experience in soccer practice; (3) non-smoking. Exclusion criteria were: (1) athletes with recent (until 6 months before the tests) osteo-myo-articular lesion which could prevent the execution of physical tests; (2) with clinical counter indication, which would prevent the performance of stress tests; (3) who were in the process of rehabilitation; (4) who play soccer as goalkeeper.

### Cardiorespiratory Test

In order to perform the maximal incremental cardiopulmonary test (MICT) with gas exchange analysis, the following protocol was selected: 3-min stimulus and 1-min of passive recovery. The initial speed of the treadmill and the respective increments per stage were: 6 km/h and 1 km/h, respectively. Regarding the protocol of the field test, the Yo-Yo Intermittent Recovery was selected with the correction especially designed for soccer athletes suggested by Castagna et al. (2006).

Ventilatory data, including the oxygen consumption (VO_2_) and the percentage of maximal oxygen consumption (%VO_2max_) were collected by the gas analyzer. The equipment was calibrated by the closed loop system. The volume sensor was calibrated using a 3L calibration syringe (Hans Rudolph). The syringe was manually handled in order to produce a flow from 0.4 to 12 L/s. The O2 and CO2 sensor was calibrated using the standard compound gas of 11.97% O2 and 4.95% original CO2, as certified by the manufacturer. The Ventilation during the test occurred through the VacuMed^®^ mouthpiece, with a nasal clip locking, just so the air would not be allowed to escape through the cavity. HR and percentage of maximal heart rate (%HR_max_) were measured during the test, and at the end of each stage for control. The blood lactate concentration was measured at the end of each stage in both tests. The temperature and relative humidity of the laboratory were controlled between 22–25°C and 50–60%, respectively.

The VT was estimated at the point where the intensity of running caused the first rise in the ventilatory equivalent of oxygen (VE/VO_2_) without a concurrent rise in the ventilatory equivalent of carbon dioxide (VE/CO2). On the other hand, the respiratory compensation point was identified by the sudden increase in the VE/CO2, according model proposed by Skinner and McLellan (1980).

During both laboratory test and field test, the rating of perceived exertion was identified by three different instruments at the end of each stage: Borg’s Ratings of Perceived Exertion 6 – 20 (Borg, 1998); Cavasini Scale (Cavasini and Matsudo, 1993); GOAL scale. Due to the details of the images, The GOAL Scale was printed on canvas in follow dimensions: 80 cm (width) × 23 cm (height). Each image on the scale was 13 cm wide × 15 cm high. The other scales were presented in laminated paper in the dimensions of 297 mm (width) × 420 mm (height).

The mouthpiece, used for gas analysis, hindered verbal communication during the test, so the athletes were instructed to point the correct anchoring of the presented instruments with their index finger of their dominant hand. The evaluator had been properly trained for the application of the test, which made it easy to observe the athlete and the classifications pointed out by them in the passive recovery periods.

Prior to the start of the test, the researchers had made the following recommendation: “We would like you to stay on the treadmill during the 3-min stimulus periods.” During the passive recovery, we would like you to put your feet on the siders and stand still. Please, use the numbers and expressions of these two scales (Borg and Cavasini Scale) as well as the numbers and images of the GOAL Scale to tell us what your body perception is during the stimulus. Look at the first image where the player is just starting the game. If you feel like this player while walking/running, your effort perception will be classified as minimal, equivalent to the number one. Now, look at the last image of this instrument. If you feel like this player instead, your perception of effort will be classified as maximum, equivalent to number six. If you feel as somewhere between the first and the last cartoon, point the image and the number which best corresponds to your level of effort.

In order to avoid some physiological interference from dehydration, the athletes ingested 5–10 ml/kg of body weight of sports drink 2 h before the test, following the ACSM Position Stand (Thomas et al., 2016).

### Instruments and Materials

The rate of oxygen consumption was measured breath by breath using an automatic ergospirometry system (V_max_ Encore 29c computerized system, SensorMedics^®^ VIASYS Healthcare Inc., Yorba Linda, CA, United States). The heart rate was measured at the end of each stage of both maximal tests using the RC3 GPS heart rate monitor, Polar^®^ (Kempele, Oulu, Finland).

The blood lactate concentration was determined by the use of the ROCHE^®^ Accutrend Plus equipment. Right at the end of each stage, the perforation of the digital pulp finger was performed, after adequate hygiene. A total of 40 microliters of blood was collected and poured into the reagent strips for the determination of [La]. Blood collection was done, in both maximal tests, by using heparinized capillaries (G100CCH, Glasscyto^®^) with 80 IU/ml of sodium heparin in order to better standardize the amount of blood in each strip. Both the lactate analyzer and the ergospirometry system were calibrated according to the manufactures’ specifications.

### Data Analysis and Statistics

The anthropometric, physiological, performance, and perceptual variables were calculated from average and standard deviation. The Shapiro–Wilk normality test was applied in order to define parametric and non-parametric variables, and their results culminated in the choice of Spearman’s linear correlation (non-parametric samples). The significance level of *p* < 0.01 was adopted in all cases. The tests were conducted by using the statistical package GraphPad Prism for Mac. The values related to the correlation force were classified according to the Hopkins et al. (2009).

## Results

**FIGURE 2:**
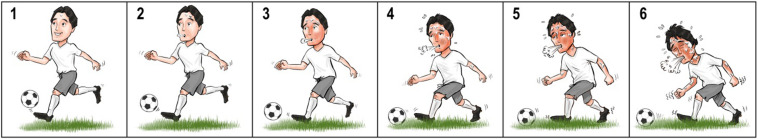
GOAL Scale. Patent registration: BR 20 2018 072989 1.

All data presented are derived from 20 soccer players Figure 2 shows the final version of The GOAL Scale after the judges’ considerations, in which it is possible to highlight: changes in ventilation signs in the different phases of exercise; biomechanical changes, including the angle of knee extension as well as the degree of trunk flexion; the incorporation of numeric anchorages in each image; as well as the use of neutral colors in the uniforms of the cartoons, thus contributing to the universalization of the instrument (Figure 2).

Outlines the values of %HR_max_, %VO_2max_, Borg RPE 6 – 20, velocity and Cavasini Scale in each anchorage of GOAL Scale during both tests (MICT and Yo-Yo Intermittent Recovery Test – Level 1) (Table 1).

**TABLE 1:** Values of psychological and physiological variables during maximal tests.

	Test	GOAL 1	GOAL 2	GOAL 3	GOAL 4	GOAL 5	GOAL 6
HR (bpm)	MICT	115.0 ± 17.0	141.0 ± 15.0	159.0 ± 16.0	173.0 ± 12.0	184.0 ± 10.0	190.0 ± 11.0
	Yo-Yo IRT1	134.0 ± 19.0	161.0 ± 20.0	180.0 ± 10.0	189.0 ± 13.0	192.0 ± 12.0	195.0 ± 10.0
%HR_max_	MICT	59.3 ± 8.5	73.0 ± 6.8	82.7 ± 7.0	90.3 ± 4.8	96.2 ± 3.2	99.0 ± 1.8
	Yo-Yo IRT1	70.4 ± 11.0	82.4 ± 10.8	93.0 ± 6.0	96.0 ± 3.2	98.9 ± 1.2	99.9 ± 0.1
RPE 6–20 (AU)	MICT	7.0 ± 1.0	9.0 ± 1.0	11.0 ± 1.0	14.0 ± 2.0	16.0 ± 2.0	19.0 ± 1.0
	Yo-Yo IRT1	7.0 ± 2.0	9.0 ± 2.0	11.0 ± 2.0	13.0 ± 2.0	16.0 ± 2.0	19.0 ± 1.0
Cavasini (AU)	MICT	1.0 ± 1.0	3.0 ± 1.0	4.0 ± 1.0	6.0 ± 1.0	8.0 ± 1.0	9.0 ± 1.0
	Yo-Yo IRT1	1.0 ± 1.0	3.0 ± 1.0	4.0 ± 2.0	6.0 ± 1.0	7.0 ± 1.0	9.0 ± 1.0
Blood Lactate (mmol/L)	MICT	2.1 ± 0.8	2.3 ± 1.0	2.8 ± 1.2	3.9 ± 1.8	6.2 ± 2.5	8.6 ± 2.8
	Yo-Yo IRT1	2.1 ± 1.0	3.6 ± 2.0	5.4 ± 2.9	8.0 ± 3.2	10.8 ± 4.2	13.9 ± 3.5
VO_2_ (ml/kg/min)	MICT	20.3 ± 5.4	32.1 ± 8.2	43.5 ± 7.6	49.9 ± 7.1	56.3 ± 6.4	60.8 ± 4.4
	Yo-Yo IRT1	37.6 ± 1.1	40.5 ± 3.4	44.0 ± 5.4	48.9 ± 3.1	52.4 ± 3.4	54.7 ± 2.8
%VO_2max_ (ml/kg/min)	MICT	33.1 ± 8.6	52.2 ± 12.9	71.3 ± 12.0	81.4 ± 9.0	92.7 ± 6.2	99.2 ± 1.5
	Yo-Yo IRT1	69.2 ± 3.9	73.1 ± 7.0	79.9 ± 10.8	87.5 ± 4.9	94.5 ± 4.1	98.9 ± 1.7
Velocity (km/h)	MICT	7.0 ± 1.0	9.0 ± 1.4	11.0 ± 1.5	13.0 ± 1.4	14.6 ± 1.4	16.0 ± 1.4
	Yo-Yo IRT1	12.0 ± 1.5	13.7 ± 1.2	14.9 ± 0.7	15.6 ± 0.6	16.3 ± 0.7	16.8 ± 0.5

*HR, heart rate; %HR_max_, percentage of maximal heart rate; RPE 6–20, Rating of Perceived Exertion 6–20 Borg Scale; VO_2_, oxygen consumption; %VO_2max_, percentage of maximal oxygen consumption; MICT, maximal incremental cardiopulmonary test; Yo-Yo IRT 1, Yo-Yo Intermittent Recovery Test Level 1; AU, Arbitrary Units; GOAL 1, GOAL Scale Anchorage 1; GOAL 2, GOAL Scale Anchorage 2; GOAL 3, GOAL Scale Anchorage 3; GOAL 4, GOAL Scale Anchorage 4; GOAL 5, GOAL Scale Anchorage 5; GOAL 6, GOAL Scale Anchorage 6.*

The correlation values among the GOAL Scale, the physiological variables (HR, %HRmax, VO_2_ e VO_2max_), and the psychophysical variables (RPE identified by different instruments) measured during MICT were: Borg RPE Scale 6 – 20 (*r* = 0.93); Cavasini Scale (*r* = 0.91); treadmill speed (*r* = 0.91); blood lactate concentration (*r* = 0.68); heart rate (*r* = 0.87); heart rate percentage (*r* = 0.91); oxygen consumption (*r* = 0.89) and oxygen consumption percentage (*r* = 0.91). All the correlation analysis was statistically significant (*p* < 0.01).

**FIGURE 3:**
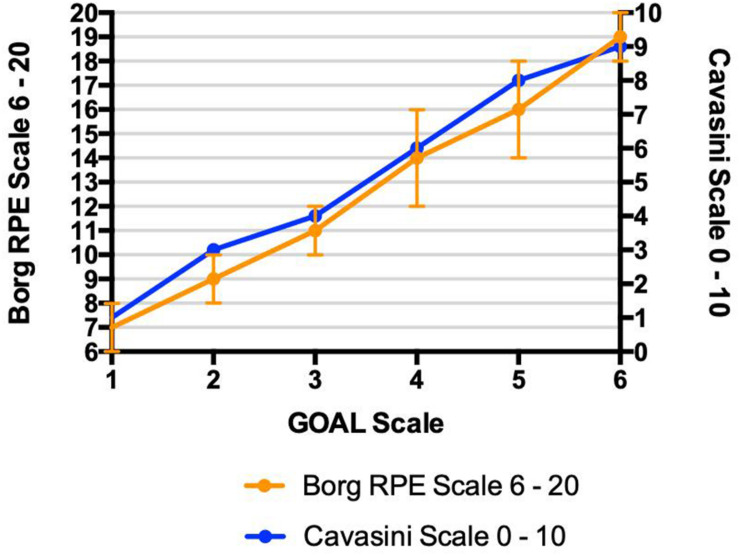
Maximal cardiopulmonary effort test – Construct validation values of Borg RPE Scale and Cavasini Scale for each value of GOAL Scale. Relationships among rating of perceived exertion identified by three different instruments (GOAL Scale, Borg RPE Scale, and Cavasini Scale) during maximal incremental cardiopulmonary test; • *r*2 = 0.86; • *r*2 = 0.83.

The construct validity of GOAL Scale during the maximal cardiopulmonary effort test, using the relationship among the scores measured on the Borg RPE Scale 6 – 20, Cavasini Scale and the GOAL Scale is demonstrated in Figure 3.

**FIGURE 4:**
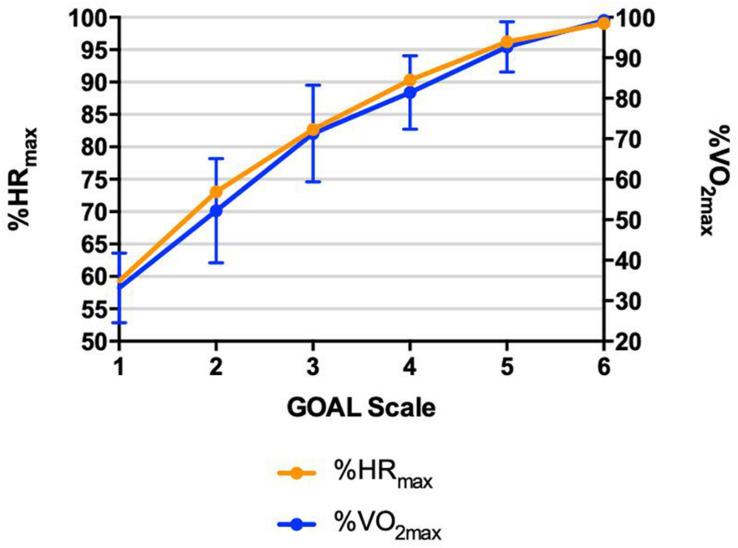
Maximal cardiopulmonary effort test – Concurrent validation relationships among rating of perceived exertion identified by GOAL Scale and physiological parameters (heart rate percentage – %HR_max_ and oxygen consumption percentage – %VO_2max_) during maximal incremental cardiopulmonary test; • *r*2 = 0.83; • *r*2 = 0.83.

The concurrent validity of GOAL Scale during the maximal cardiopulmonary effort test, using the relationship among the scores measured on the GOAL and the respective values measured in physiological parameters is demonstrated in Figure 4.

The correlation values into GOAL Scale and other physiological and psychophysical variables measured during the field test were: Borg RPE Scale 6– 20 (*r* = 0.88); Cavasini Scale (*r* = 0.90); stage speed (*r* = 0.89); blood lactate concentration (*r* = 0.83); heart rate (*r* = 0.83); heart rate percentage (*r* = 0.86); oxygen consumption (*r* = 0.88) and oxygen consumption percentage (*r* = 0.83). All the correlation analysis was statistically significant (*r* < 0.01).

**FIGURE 5:**
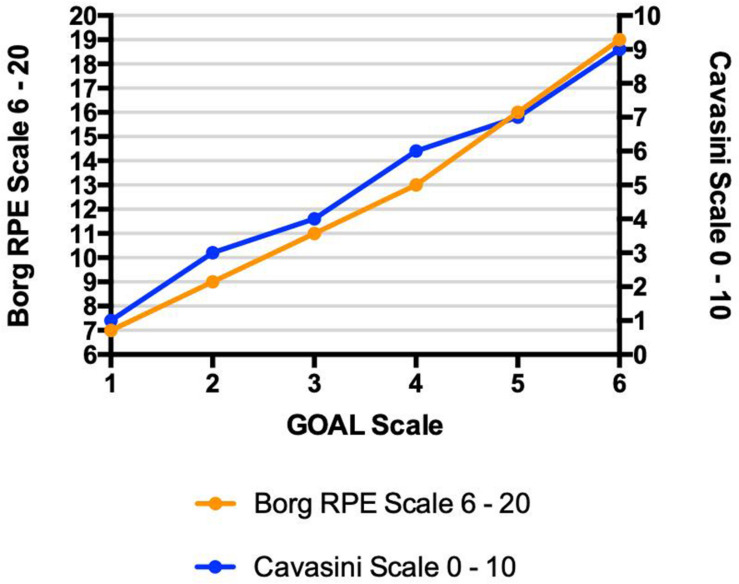
Yo-Yo Test – Construct Validation Relationships among rating of perceived exertion identified by three different instruments (GOAL Scale, Borg RPE Scale, and Cavasini Scale) during Yo-Yo Intermittent Recovery Test – Level 1; • *r*2 = 0.77; • *r*2 = 0.81.

The construct validity of GOAL Scale during the field test, using the relationship among the scores measured on the Borg RPE Scale 6–20, Cavasini Scale and the GOAL Scale is demonstrated in Figure 5.

**FIGURE 6:**
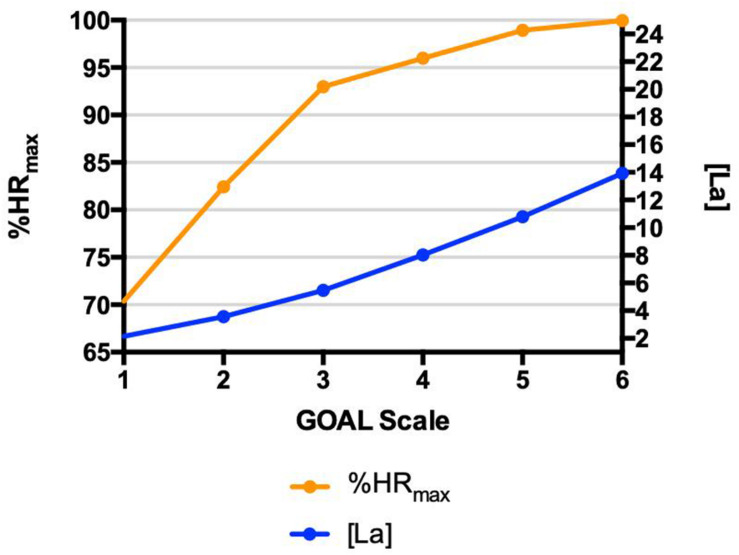
Yo-Yo Test – Concurrent Validation Relationships among rating of perceived exertion identified by GOAL Scale and physiological parameters (heart rate percentage – %HR_max_ and blood lactate concentration) during Yo-Yo Intermittent Recovery Test – Level 1; • *r*2 = 0.73; • *r*2 = 0.69.

The concurrent validity of GOAL Scale during the Yo-Yo Intermittent Recovery Test – Level 1, using the relationship among the scores measured on the GOAL and the respective values measured in physiological parameters is demonstrated in Figure 6.

**FIGURE 7:**
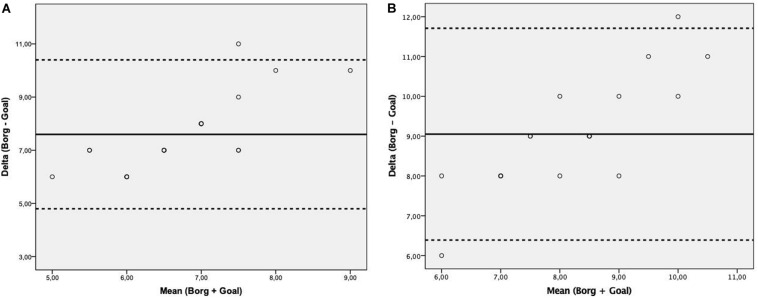
Bland-Altman plot assessing the agreement between the Borg 6–20 scale and the Goal scale at ventilatory threshold **(A)** and respiratory compensation point **(B)** during cardiorespiratory test. Solid line represents the mean difference (bias) between Borg 6–20 and Goal scale. Upper and lower dashed lines represent the 95% limits of agreement (mean difference ± 1.96 × standard deviation of the difference).

In order to prescribe training sessions is very important to know the ventilatory threshold (VT) and respiratory compensation point (RCP) and the corresponding values of perceived exertion. (Table 2) shows the values of different instruments of perceived exertion which corresponding with the VT and RCP. The Figure 7 shows the agreement between the Borg 6 –20 scale and the GOAL scale at ventilatory threshold and respiratory compensation point during cardiorespiratory test.

**TABLE 2:** Ventilatory threshold and respiratory compensation point and their corresponding variables.

Variable	MICT RCP	MICT VT	Yo-Yo IRT1 RCP	Yo-Yo IRT1 VT
GOAL	3.0 ± 1.0	4.0 ± 1.0	2.0 ± 1.0	3.0 ± 1.0
RPE 6–20	11.0 ± 2.0	13.0 ± 2.0	9.0 ± 2.0	10.0 ± 2.0
CAVASINI	4.0 ± 1.0	5.0 ± 2.0	2.0 ± 2.0	1.0 ± 2.0
%HR_max_	80.8 ± 3.8	89.4 ± 3.1	80.4 ± 4.3	88.8 ± 4.3
%VO_2max_	68.0 ± 6.8	80.5 ± 4.1	71.2 ± 3.6	76.4 ± 3.9
[La]	2.3 ± 0.9	3.5 ± 1.0	2.5 ± 1.0	3.7 ± 1.4

*GOAL, GOAL Scale; RPE 6–20, Rating of Perceived Exertion 6–20 Borg Scale; CAVASINI, Cavasini Scale; %HR_max_, percentage of maximal heart rate; %VO_2max_, percentage of maximal oxygen consumption; [La], blood lactate concentration; MICT RCP, respiratory compensation point in the maximal incremental cardiopulmonary test; MICT VT, ventilatory threshold in the maximal incremental cardiopulmonary test; Yo-Yo IRT 1 RPC, respiratory compensation point in the Yo-Yo Intermittent Recovery Test Level 1; Yo-Yo IRT 1 VT, ventilatory threshold in the Yo-Yo Intermittent Recovery Test Level.*

As the frequency of use of the anchorages of each instrument was evaluated, in the TECM and in the Yo-Yo Intermittent Recovery Test – Level 1, the results found were, respectively: 94.2 ± 11.18% and 89.2 ± 15.56% of the total of the GOAL Scale anchorages (six in all); 76.8 ± 14.89% and 64.1 ± 15.18% of the total of the Cavasini Scale (11 in all); 58.7 ± 12.35% and 49.7 ± 12.70% da Borg 6 – 20.

## Discussion

The aim of this research was to elaborate and to validate a specific pictorial perceived exertion scale for soccer players. The validity process used in this research has verified the association into RPE values found by GOAL Scale and the other physiological and psychophysiological variables during laboratory and field progressive tests. The results showed significant correlation values which make it able to demonstrate the validity of the instrument and, from this reality, to suggest its use as an intensity control instrument on athletes of this modality.

On the other hand, it is necessary to highlight the non-linear relationship among GOAL Scale and physiological variables that was found. According to Stevens (1956) and psychophysical power law, sensations grow as a power of stimulus that is different according to different types of stimulus (Gescheider, 1997). Thusly, the increase in the perceptual response to growing stimuli of work rate, when it is related with physiological (and objective) parameters (cited above) has been known to follow a non-linear path (Borg et al., 1987; Pincivero et al., 2003).

In the scientific validity of RPE Borg scale (instrument most frequently discussed in the scientific literature), the association with objective physiological variables (heart rate and oxygen consumption) was evaluated in 40 healthy subjects, which supports the method used in the instrument validation for soccer players (Borg and Kaijser, 2006), in which the results demonstrated positive association between the rating of perceived exertion, identified by GOAL Scale, HR, %VO_2max_, [La], RPE (identified by Borg and Cavasini Scale), in both tests (laboratory and field). The ratings and heart rates were recorded every minute and blood lactates every third minute during the work tests.

In attempt to elaborate more specific instruments, perceived exertion OMNI scales were validated for different sports and publics, including resistance training (Robertson et al., 2003), cycloergometer training (Robertson et al., 2004) and training for children (Robertson et al., 2005). Similarly to what was performed in Borg’s study and in GOAL scale validation, the studies mentioned above also used the correlational method (between objective and subjective parameters) for measurement of instruments’ scientific validity. Recently, Micklewright et al. (2017) developed and validated the Fatigue Classification Index, instrument elaborated from numerical, verbal, and diagrammatic anchorages which has the purpose to evaluate the perceived fatigue. The authors carried out four experiments involving eighteen healthy adult males, similarly to the present research. Another point is that the authors analyzed the same physiological variables that were analyzed in the present research, including: heart rate, breath-by-breathe ventilation and gas measurements and blood lactate concentration, which confirm the validity of these variables as objective variables in order to confirm the validity of the effort subjective variables through their relationship.

The perceived exertion is a variable frequently used to monitor the training load which the soccer athletes (indoor and field) are submitted to throughout a season (Impellizzeri et al., 2005; Casamichana et al., 2013; Moreira et al., 2013). It is possible to affirm that one great advantage of this instrument is its sensibility to different parameters which are present in all training programs, including intensity (Connolly and Tenenbaum, 2010; Bourdon et al., 2017), volume, exercise type (Thorpe et al., 2016), level of involved participants and their psychological attributes (Hutchinson et al., 2008). Following this train of thought, Gaudino et al. (2015) evaluated twenty-two elite players competing in the English Premier League, and showed that volume variables (sprints and the number of contacts and acceleration actions) can exert straight influence on the RPE values signaled by the soccer players. Therefore, it is possible to affirm that the perceived exertion is a variable which is sensible to different workouts organization and that it can integrate both physiological and psychological variables, still respecting the players’ individuality.

In spite of the fact that the RPE is the most frequently used instrument to measure the effort in soccer and futsal players, Polito et al. (2017) highlighted that the subjective data achieved with its use should be continuously associated with other objective measured parameters during all season, including heart rate, blood lactate concentration, traveled distance, number of contacts in plyometric training, which promote the guarantee of a better data interpretation by physical trainers, coaches and psychologists aiming a greater (re)orientation of the training sessions. Analyzing a professional female futsal team over 45 sessions, Milanez et al. (2014) evaluated the relationship between the internal training load (calculated by the perceived exertion method) and salivary immunoglobulin A (SIgA) and the data found shows that the training load above 435 AU (arbitrary units) is associated with decrease of immunity, providing one more possible use of the perceived exertion in this sport.

However, despite the strong consistence of the perceived exertion instruments, Impellizzeri et al. (2011) asked attention to three different situations, in which the RPE could present greater intersubjective variability: (1) when developed without psychophysical proceedings needed; (2) when modified without proper care to keep the original characteristics of the instrument, especially during the translation process; (3) when proper correspondence with exercise intensity indicators is not shown.

Therefore, the simple translation of perceived exertion scales to different languages without the proper methodological proceedings (cross-cultural validation, for example), which allows the maintenance of its original properties, is a risk for its validity and for the collected data obtained from it as well, which can be avoided with cartoon scales.

Another factor observed in the present research was the relationship between the cartoons and the anaerobic thresholds, by using the Multiple Threshold Model in different exercise phases as proposed by Skinner and McLellan (1980). The results showed that the cartoons numbers 3 and 4 of GOAL Scale corresponded, respectively, to thresholds 1 and 2 in MICT. On the other hand, in the field test, the first and second threshold were related to the cartoons numbers 2 and 3 of GOAL Scale, respectively. It was found the same trend for the other scales, which demonstrated greater values to the same relative submaximal load in MICT when compared to the field test, which can be reinforced by the scientific literature, which affirms greater RPE values in laboratorial conditions, as these conditions are less specific situations to the real practice of the sports in general (St Gibson et al., 2006). These findings contribute to the understanding of the sensibility showed by athletes in perceived exertion identification in different situations.

Some noteworthy limitations must be addressed when interpreting or applying these results: (1) the study only evaluated the application of the GOAL Scale to measure the perceived exertion of soccer athletes in different maximal effort tests, therefore, special care is needed when using the GOAL Scale for other sports and for other variables before testing it; (2) the interrelationships between the different anchors of the scale were not evaluated, which should be the next researches involving this pictorial instrument.

The perception of effort is an essential variable to measure the loads applied during the training program for soccer, futsal and beach soccer players. In order to avoid the wrong translation by players of different languages, or misinterpretation by players of different literacy levels, the GOAL Scale is a good alternative to be applied to athletes of these modalities.

This research provides firm support for the use of the GOAL Scale as an instrument able to estimate the intensity of effort that the beach soccer, futsal, and soccer players realize through subjective perception, contributing with the effort intensity control. It is suggested that the next steps would be the reliability evaluation of the GOAL Scale, and the use of the GOAL Scale in the real context of the beach soccer, futsal and soccer games, identifying how the data provided by this instrument relates to the external load indicators.

## Data Availability Statement

The raw data supporting the conclusions of this article will be made available by the authors, without undue reservation.

## Ethics Statement

The studies involving human participants were reviewed and approved by the Comitê de Ética em Pesquisa Universidade São Judas Tadeu. Written informed consent to participate in this study was provided by the participants’ legal guardian/next of kin.

## Author Contributions

LP and MB conceived the original idea of the study. MV selected the researches and contributed to data processing and analysis. LP and DM analyzed and presented the data. LP and MM wrote and organized the manuscript. All authors reviewed the document and approved the final version for submission.

## Conflict of Interest

The authors declare that the research was conducted in the absence of any commercial or financial relationships that could be construed as a potential conflict of interest.
